# Gut resistome development in healthy twin pairs in the first year of life

**DOI:** 10.1186/s40168-015-0090-9

**Published:** 2015-06-25

**Authors:** Aimee M. Moore, Sara Ahmadi, Sanket Patel, Molly K. Gibson, Bin Wang, Malick I. Ndao, Elena Deych, William Shannon, Phillip I. Tarr, Barbara B. Warner, Gautam Dantas

**Affiliations:** Department of Pediatrics, Washington University in St Louis School of Medicine, 660 S. Euclid Avenue, St. Louis, MO 63110 USA; Department of Pathology and Immunology, Washington University in St. Louis School of Medicine, 660 S. Euclid Avenue, St. Louis, MO 63110 USA; Center for Genome Sciences and Systems Biology, Washington University in St. Louis School of Medicine, 4444 Forest Park Boulevard, St. Louis, MO 63108 USA; Department of Biostatistics, Washington University in St. Louis School of Medicine, 660 S. Euclid Avenue, St. Louis, MO 63110 USA; Department of Molecular Microbiology, Washington University in St. School of Medicine, 660 S. Euclid Avenue, St. Louis, MO 63110 USA; Department of Biomedical Engineering, Washington University in St. Louis, One Brookings Drive, St. Louis, MO 63130 USA

**Keywords:** Antibiotic resistance, Gut microbiome, Fecal microbiome, Pediatrics, Beta lactamase, Chloramphenicol resistance

## Abstract

**Background:**

The early life of the human host marks a critically important time for establishment of the gut microbial community, yet the developmental trajectory of gut community-encoded resistance genes (resistome) is unknown. We present a longitudinal study of the fecal antibiotic resistome of healthy amoxicillin-exposed and antibiotic-naive twins and their mothers during the first year of life.

**Results:**

We extracted metagenomic DNA (mgDNA) from fecal samples collected from three healthy twin pairs at three timepoints (1 or 2 months, 6 or 7 months, and 11 months) and from their mothers (collected at delivery). The mgDNA was used to construct metagenomic expression libraries in an *Escherichia coli* host. These libraries were screened for antibiotic resistance, and functionally selected resistance genes were sequenced and annotated. A diverse fecal resistome distinct from the maternal resistome was apparent by 2 months of age, and infants’ fecal resistomes included resistance to clinically important broad-spectrum beta-lactam antibiotics (e.g., piperacillin-tazobactam, aztreonam, cefepime) not found in their mothers. Dissemination of resistance genes among members of a given family was positively correlated with sharing of those same resistance genes between unrelated families, potentially identifying within-family sharing as a marker of resistance genes emerging in the human community at large. Finally, we found a distinct developmental trajectory for a community-encoded function: chloramphenicol resistance. All study subjects at all timepoints harbored chloramphenicol resistance determinants, but multidrug efflux pumps (rarely found in mothers) were the primary effectors of chloramphenicol resistance in young infants. Chloramphenicol acetyltransferases were more common in mothers than in infants and were found in nearly all the infants at later timepoints.

**Conclusions:**

Our results suggest that healthy 1–2-month-old infants’ gut microbes harbor clinically relevant resistance genes distinct from those of their mothers, and that family-specific shared environmental factors early in life shape resistome development.

**Electronic supplementary material:**

The online version of this article (doi:10.1186/s40168-015-0090-9) contains supplementary material, which is available to authorized users.

## Background

The human gut microbiota is an important reservoir of antibiotic resistance genes (resistome), which can be exchanged with pathogens [1–4]. Diverse antibiotic resistance genes are present in the gut microbiota of young infants soon after birth, even without antibiotic therapy [5–12]. While the overall architecture of the gut microbial community undergoes chaotic shifts in the first years of life before stabilizing towards an adult-like state [13–16], the trajectory and dynamics of resistome development in healthy young infants are poorly understood. Children in the United States are heavily antibiotic-exposed [11, 12], with unclear effects on resistome development. In this study, we present a longitudinal functional metagenomic interrogation of gut resistome development during the first year of life in three pairs of healthy, vaginally delivered, formula-fed twins enrolled in the St. Louis Neonatal Microbiome Initiative. One pair was antibiotic-naïve for the entire sampling interval, one pair was concordant (receiving 10 days of amoxicillin simultaneously at 8 months of age) and one was discordant (one sibling received 10 days of amoxicillin at 8 months and the other did not).

## Results

**Fig. 1:**
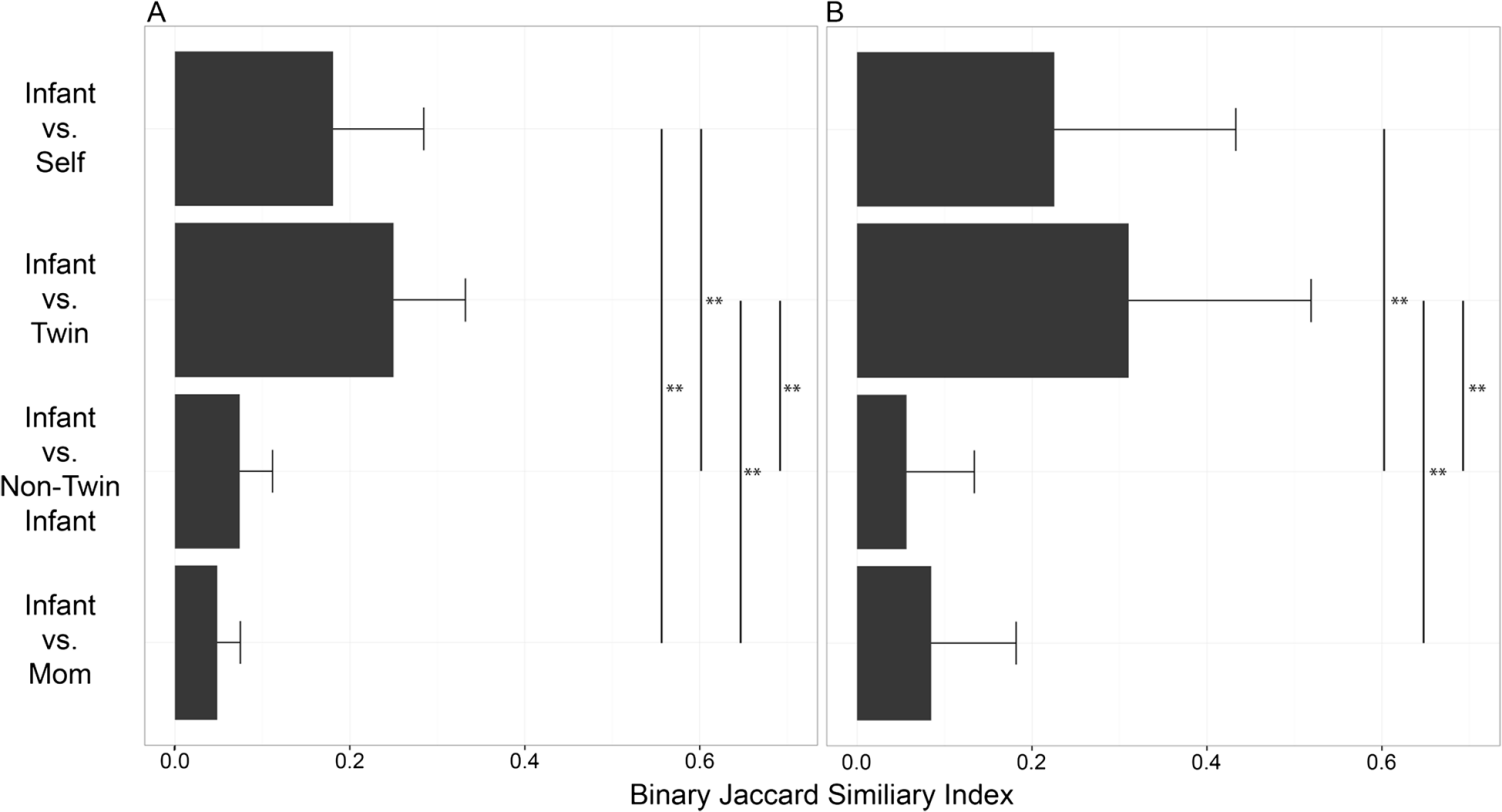
Twin infant fecal resistomes resemble those of their siblings. Predicted resistance proteins were collapsed into 97 % identity clusters. Binary Jaccard resistance protein cluster composition similarity was determined for (1) the same infant at different timepoints (self-sharing), (2) twin siblings, (3) unrelated infants, and (4) mothers and infants from the same family. **a** All resistance proteins at *left*; **b** the subset of β-lactamases and penicillin-binding proteins at *right*. Significance was calculated using the Student’s *t*-test with 1000 Monte Carlo simulations (**p < 0.01). Infant resistomes overall (**a**) were significantly more similar to a twin sibling or to the same subject at different timepoints than to their mothers or unrelated infants. Infant resistomes were no more similar to those of their mothers than to unrelated infants. There was also no significant difference between the similarity between infants and their twin sibling and the persistence of resistance proteins within a given individual at different timepoints (self-sharing)

Demographic information regarding the three families included in this study is listed in Additional file 1: Table S1. We constructed 21 fecal metagenomic libraries averaging 9.3 ± 7.9 (mean ± s.d.) gigabases (Gb) in size, from 18 infant samples and 3 maternal samples, and used functional metagenomic selections paired with next-generation sequencing to identify a total of 905 unique predicted antibiotic resistance protein clusters (collapsed at 97 % amino acid identity using cd-hit [17, 18]). In all individuals, at all timepoints, we identified protein clusters conferring resistance to five different antibiotic classes (β-lactams, tetracycline, chloramphenicol, trimethoprim, and cycloserine). Proteins conferring resistance to aminoglycosides, tigecycline, and colistin were also found, but less frequently. Of the 905 resistance protein clusters identified, 296 (32 %) were unique to mothers, 531 (59 %) were unique to infants, 142 (15.6 %) were shared between twin siblings, 159 (17.5 %) persisted within the same individual at multiple timepoints, and 106 (11.7 %) were shared between unrelated infants. Only 51 (5.6 %) were shared between infants and their mothers. Twin siblings’ resistomes were significantly more similar to each other than to those of their mothers or unrelated infants (*p* < 0.01, Student’s *t*-test with 1000 Monte Carlo simulations, Fig. 1). Conversely, they were as similar to their siblings’ resistomes as they were to their own at other points in time and no more similar to their mothers’ resistomes than to unrelated infants’ resistomes (Fig. 1). There was no difference in sharing between infants and their own mothers and infants and unrelated mothers. When sharing of resistance proteins between siblings was separated by individual timepoint, infant resistomes were significantly more similar to their twin sibling’s resistome than to the resistome of an unrelated infant (*p* = <0.005, all timepoints, Additional file 1: Figure S3). The composition of infant resistomes appears generally robust in the face of amoxicillin perturbation: neither the overall population of resistance protein nor the specific sub-population of β-lactamases was affected by amoxicillin exposure when measured approximately 3 months after antibiotic treatment. There was, however, a statistically significant increase in the number of multidrug efflux pumps conferring resistance to β-lactam antibiotics at 3 months after amoxicillin exposure in these infants (*p* < 0.0001, log-linear model, Additional file 1: Table S3).

**Fig. 2:**
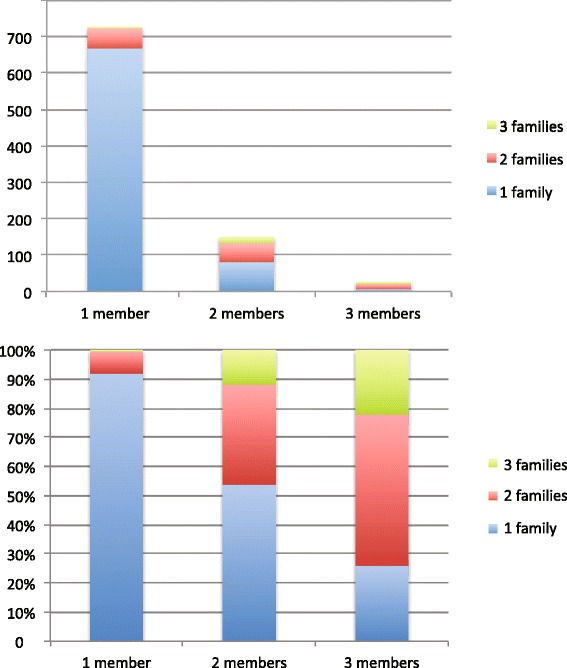
Sharing of resistance-associated proteins within and between families. The *top* graph shows absolute counts of resistance-associated protein clusters, grouped by the number of members within a given family they were identified in. Most protein clusters were only identified in one member of one family; much smaller numbers were identified in multiple family members or multiple families. The *lower* graph shows the proportions of resistance-associated protein clusters identified in multiple families, grouped by the number of members of a single family they were identified in. Larger proportions of resistance-associated proteins that are shared are multiple members of a single family are also identified in multiple unrelated families

We used a log-linear model to test the likelihood that resistance-associated protein clusters would be shared within multiple members of a given family, or between unrelated families (Fig. 2). Although resistance-associated proteins were less likely to be found in more than one family member than in just one individual (*p* < 0.0001, Additional file 1: Table S4) and less likely to be found in multiple families than in a single family (*p* < 0.0001, Additional file 1: Table S4), resistance proteins that were found in more than one individual within a family were also significantly more likely to be found in more than one family (*p* < 0.0001, Additional file 1: Table S4). We also used a log-linear model to test the likelihood that resistance-associated protein clusters that persisted within a given individual at different timepoints were more likely to be shared within and between families. Although resistance proteins were significantly less likely to be persistent at multiple timepoints than found at a single timepoint (*p* < 0.0001, Additional file 1: Table S10), and also less likely to be found in multiple individuals than in a single individual (*p* < 0.0001, Additional file 1: Table S10), resistance proteins that persisted within an individual over time were also significantly more likely to be found within multiple members of the same family (*p* = 0.0449). There was no significant interaction between persistence of resistance proteins within an individual over time and sharing between unrelated families.

We used a log-linear model to test the contribution of co-localization with a mobile genetic element to sharing of resistance protein clusters within members of a single family and between unrelated families. One hundred thirty-six of 905 (15.1 %) resistance-associated protein clusters were encoded by genes co-localized with a mobile genetic element. However, this co-localization was not significantly associated with sharing of a protein cluster within members of a given family (*p* = 0.2212, Additional file 1: Table S5) or between members of unrelated families (*p* = 0.2655, Additional file 1: Table S5).

**Fig. 3:**
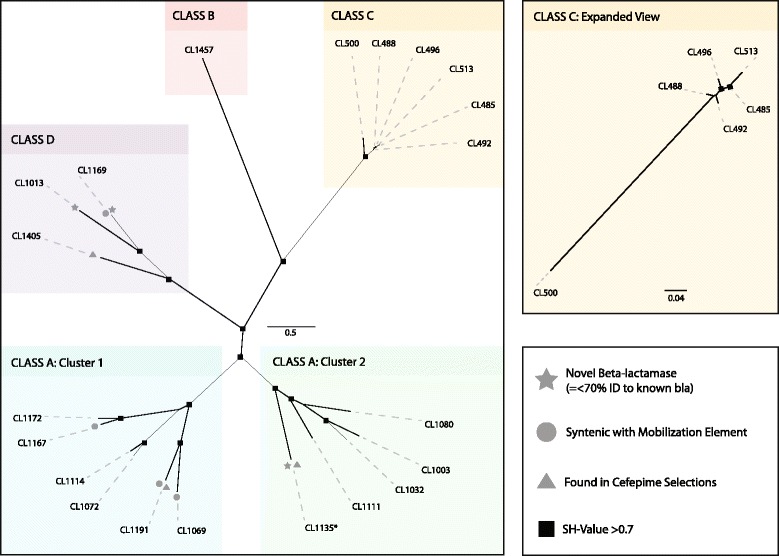
β-lactamase phylogenetic tree. Predicted β-lactamase protein sequences were collapsed into 97 % ID clusters. All β-lactamase protein sequences with at least 90 % coverage of the nearest hit in the NCBI nr database were included in the tree. Multiple alignment was done with Muscle and the tree was made using FastTree. Nodes with an S-H value >0.7 are marked with a *square*. All classes of β-lactamases are present. Class A β-lactamases separated into two groups: one with high identity to TEM extended-spectrum β-lactamases and one without. β-lactamases co-localized with mobile genetic elements are marked with a *gray dot*. Novel β-lactamases with less than 70 % identity to any known β-lactamase are marked with a *star*. β-lactamases found in cefepime selections are marked with a *triangle*

**Fig. 4:**
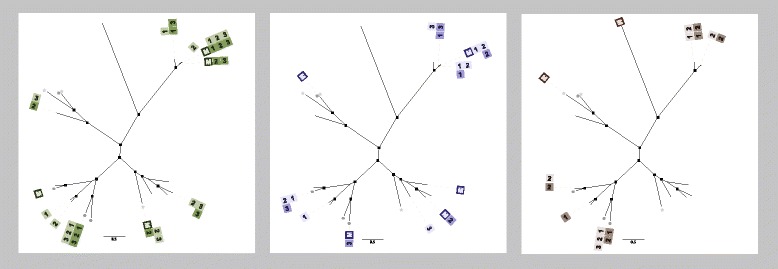
Populations of chloramphenicol resistance proteins change over time. Predicted proteins found when fecal metagenomic libraries were screened on chloramphenicol-containing media were collapsed into 97 % ID clusters. *Black boxes* signify genes encoding a resistance protein that were identified in the fecal metagenome of a study subject at a given timepoint, while *white* or *light gray squares* indicate that the protein was not present. Proteins that were co-localized with a mobile genetic element are marked with an *asterisk*. Chloramphenicol acetyltransferases were found in all mothers and in five of the six infants at the final timepoint, but were qualitatively less common in infants at earlier timepoints. By contrast, multidrug efflux pumps were rare in mothers and in 11-month-old infants, but were commonly found in earlier samples

β-lactam resistance was universal across the study cohort, yet there was substantial inter-subject variation in the pattern of specific β-lactam resistance phenotypes (Figs. 3 and 4 and Additional file 1: Figures S1 and S2). Only 5 of 47 (10.7 %) β-lactamase family proteins were shared between infants and mothers, similar to the proportion shared with unrelated infants (4 of 47, or 8.5 %). Consistent with trends observed in the general resistome, β-lactam resistance patterns were similar between siblings and stable over time, where 11 of 47 (23.4 %) β-lactamases and penicillin-binding proteins were shared with the infants’ twin sibling and 13 of 47 (27.7 %) persisted within the same individual at multiple samplings. Infant β-lactam resistomes were significantly more like their own resistome at another timepoint or a twin sibling’s resistome than the resistome of an unrelated infant, and significantly more similar to their twin siblings’ than to their mothers’ resistomes (*p* < 0.01, Student’s *t*-test with 1000 Monte Carlo simulations, Fig. 1b). When each timepoint was analyzed separately, twin siblings’ resistomes were only significantly more similar to each other than to those of an unrelated infant of the same age at timepoint 2 (Additional file 1: Figure S3).

Chloramphenicol resistance was present in all subjects at all timepoints. We observed distinct infantile and adult-like populations of chloramphenicol resistance genes. In the fecal samples collected in early infancy (1 or 2 months, and 6 or 7 months), chloramphenicol resistance genes primarily encoded multidrug efflux pumps (Fig. 2). We used a log-linear model to test the likelihood that multidrug efflux pumps were found in mothers versus infants and that these pumps became less common over time. Multidrug efflux pumps were significantly more common in infants than in mothers (*p* = 0.0009, Additional file 1: Table S6) and were significantly less likely to be found as infant age increased during the first year of life (*p* < 0.0001, Additional file 1: Table S7). A majority (85 %) of these efflux pumps had >90 % identity to efflux pumps of Enterobacteriaceae origin; only one (CL2137) was identical to a bifidobacterial major facilitator transporter (WP_003817038). Some multidrug efflux pumps originated from source contigs with sequences identical to those of bona fide pathogens: a 3737 bp contig from a 1-month-old infant (and a 1460 bp contig from the twin sibling) had 100 % sequence identity to the recently described pan-resistant *Enterobacter aerogenes* strain EA1509E [19].

While multidrug efflux pumps were the most common effectors of chloramphenicol resistance in stools of young infants, chloramphenicol acetyltransferases were more frequently associated with maternal resistomes. We used a log-linear model to test the likelihood that chloramphenicol acetyltransferases were found in mothers versus infants and that these acetyltransferases became more common over time. Chloramphenicol acetyltransferases were significantly more common in mothers than infants (*p* < 0.0001, Additional file 1: Table S8), were rarely found in the youngest infants, but were found in five of the six infants at the final (11-month) timepoint (Fig. 4). There was, however, no significant increase over time in the likelihood of finding chloramphenicol acetyltransferases in infant gut microbiomes (*p* = 0.2930, Additional file 1: Table S9). Twenty-five percent of the chloramphenicol acetyltransferases identified in this study population were co-localized with mobile genetic elements. Novel proteins made up more than half of the chloramphenicol acetyltransferases identified in this study: seven of the 12 chloramphenicol acetyltransferase clusters identified had <75 % protein identity to any known acetyltransferase. The source contigs for these novel resistance proteins also had low identity to any known organism.

## Discussion

In this study population, we found a diverse fecal resistome established within 1–2 months of birth. Prior microbiome studies of twin infants have shown that siblings’ microbiomes undergo similar changes over time [13] and are similar to each other irrespective of zygosity [15, 20, 21]; the patterns of sharing of resistance-associated proteins between twin siblings we observed are consistent with this prior work on the overall microbial community, and likely reflect similarities between siblings in microbial community composition. We also found that infants’ gut resistomes differed from those of their mothers in early life, which is also consistent with studies showing that the gut microbiota of twin infants are more similar to each other than to their mothers’ microbiota [20] and that infant gut-associated tetracycline resistance genes [6, 7] are distinct from those in the maternal fecal microbiota [6, 7]. The assembly of a diverse gut resistome within the first 2 months of life, and the greater resemblance of infant gut resistomes to their own and their siblings’ resistomes at other timepoints than to the resistomes of unrelated infants, suggest that family-specific shared environmental factors early in life shape resistome development. The dissimilarity of infant resistomes to those of their mothers suggests that, although gut microbes may be shared between the mothers and vaginally delivered infants [22], the maternal gut resistome is not the primary driver of gut resistome establishment in infants. The previously observed pattern in which twin siblings’ microbiomes are similar to each other and dissimilar to their mothers’ microbiome also applies to the gut resistome, suggesting that the infants’ gut resistome is likely a reflection of community composition.

**Fig. 5:**
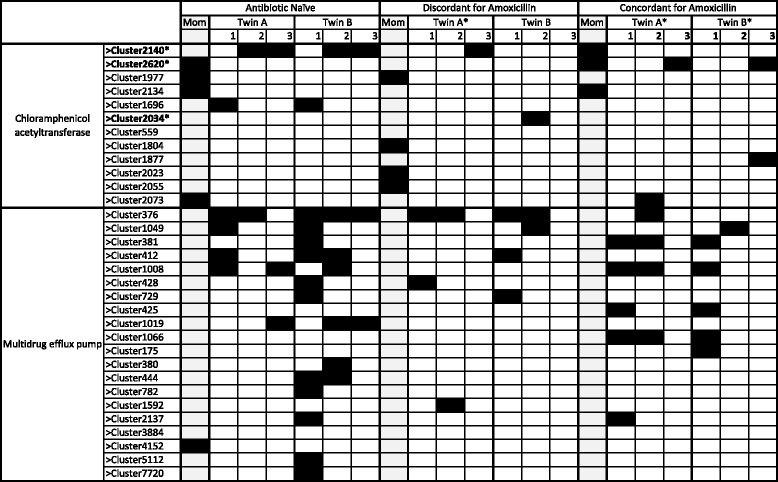
β-lactam phylogenetic tree, annotated by study subject. Maternal subjects are marked with an “M”. Infant fecal samples are marked with a number; “1” indicating the first (baseline) sample collected at 1–2 months of age, “2” indicating the second sample collected at 6–7 months of age, and “3” indicating the third sample collected at 11 months of age. The antibiotic-naïve control family is colored *green*, the family with infants discordant for amoxicillin exposure at 8 months of age is colored *purple*, and the family with infants concordant for amoxicillin exposure at 8 months of age is colored *brown*. Infant twin A subjects are *shaded darker*; twin B subjects are *shaded lighter*. β-lactamases were commonly present in both members of a twin pair, and frequently persisted at more than one timepoint within a given subject. Many β-lactamases identified in the infant fecal microbiomes were not present in the maternal microbiome

In every family, phenotypic resistance to at least one broad-spectrum β-lactam encoded by an infant gut resistome was not observed in the mothers’ resistome, although the specific antibiotics involved (piperacillin-tazobactam, cefotaxime, aztreonam, and/or cefepime) varied (Figs. 3 and 5 and Additional file 1: Figure S1). The genetic dissimilarity between infants’ beta-lactam resistomes and those of their mothers likely explains this phenotypic difference, as phylogenetic groups of resistance genes were often associated with specific β-lactam antibiotics. For example, one group of class A β-lactamases with high identity to TEM β-lactamases was associated with extended-spectrum penicillin and β-lactamase inhibitor resistance (Fig. 3 and Additional file 1: Figure S2), while another class A group was more closely associated with resistance to later-generation cephalosporins. The origin of the broader-spectrum beta-lactam resistance in infant gut resistomes is unclear, although colonization with antibiotic-resistant hospital bacterial strains is a possibility that warrants further study (all newborn subjects were healthy hospital-born infants). However, we wish to note that the multidrug-resistant pathogen *Escherichia coli* ST131 does not colonize the infants in this cohort until well after discharge from the hospital [23]. The lack of time-dependent effects on β-lactam resistance genes despite inter-subject variation in exposure to amoxicillin may reflect the sampling resolution in this small study, in which the resistome is sampled 1 or 2 months prior to antibiotic therapy and 3 months after antibiotic exposure. Prior studies have shown acute antibiotic-induced perturbations in adult gut microbial community concomitant with host antibiotic exposure, followed by partial recovery [24, 25]. The binary data regarding presence or absence of resistance genes generated by this study also precludes detection of any changes in the relative abundance of certain taxa following antibiotic exposure. Future studies including more frequent fecal sampling surrounding the time of host antibiotic exposure and additional data regarding the abundance of selected resistance genes may illuminate more acute and transient antibiotic effects that our study was not designed or powered to detect.

Understanding patterns of gut-associated resistance gene dissemination within human communities has epidemiologic implications. We found that sharing of resistance genes within families was positively associated with sharing between families, and that persistence of resistance genes within an individual over time was positively associated with sharing with within-family sharing, but not with between-family sharing. Co-localization with a mobile genetic element was not significantly correlated with identification of resistance proteins in multiple individuals. This finding may be explained by undercounting mobilizable elements in this functional metagenomic study, where nearly all contigs identified were <5 kilobases (kb). This concern is partially mitigated by the demonstration that trends in resistance gene and mobilization element co-localization predicted by small-insert functional metagenomics are recapitulated by matched calculations from bacterial whole genome sequences [26]. Alternatively, our data may suggest that the distribution of gut-associated resistance genes primarily reflects the prevalence of specific host bacteria within the human community, rather than any association with mobile genetic elements, as has been demonstrated in soil microbiomes and resistomes [26]. The positive association between persistence within individuals over time and within-family sharing but not between-family sharing may be a reflection of host genetics or shared environment shaping microbial communities; microbes that are more likely to persist within an individual may also be more likely to colonize close relatives. The potential role for examining genes shared within families to detect clinically important resistance genes emerging human communities warrants further investigation.

Chloramphenicol resistance was universal in all subjects at all timepoints, consistent with our previous resistome study of single timepoint samples of unrelated infants and children [5] as well as with prior studies showing highly prevalent chloramphenicol resistance in adult populations [27]. The reason for universal chloramphenicol resistance is unclear. Agricultural use of chloramphenicol providing selection pressure (the “farm to fork” model [27]) is a potential factor; alternatively, chloramphenicol resistance genes may play a yet-to-be determined role as a core functional component of the gut microbiome [20]. Chloramphenicol resistance genes demonstrated a developmental progression from a neonatal to an adult-like state. The infant chloramphenicol resistome was initially dominated by multidrug efflux pumps, which were uncommonly found in maternal chloramphenicol selections, and became less common over time. Because most of the source contigs for these multidrug efflux pumps had high identity to Enterobacteriaceae, it is unlikely that the predominance of multidrug efflux pumps in early infancy is a result of bifidobacteria- or lactobacilli-enriched infantile gut microbial communities. Importantly, our past studies with adult and pediatric human microbiome samples have demonstrated functional capture of numerous resistance genes of Firmicutes, Bacteroidetes, and Actinobacteria origin, without over-representation of Proteobacterial genes [5, 28], suggesting the observed Enterobacteriaceae multidrug efflux pump enrichment signal is not an artifact of cloning bias in our *E. coli* screening host. Prior studies have shown that Enterobacteriaceae are enriched in the gut microbial communities of young infants and decrease with time [15, 29]; the observed decrease in chloramphenicol-resistant multidrug efflux pumps during the first year of life in this cohort may be a reflection of maturation of the gut microbial community. Chloramphenicol acetyltransferases were more commonly found in mothers, although they were identified in all infant subjects at some timepoint. This finding of cryptic organisms as an important source of novel chloramphenicol resistance genes is consistent with our prior survey of the antibiotic resistome of healthy children [5].

## Conclusions

This longitudinal study of three pairs of healthy twins and their mothers shows that a diverse gut resistome is present in the first 2 months of life. This confirms our prior hypothesis that multiple antibiotic resistance is present in very young infants [5], and underscores the importance of future study to understand the environmental determinants of resistome establishment in the first weeks of life. Prior studies have shown that gut microbial communities and community-encoded microbial functions of twin siblings are similar to each other [13, 15, 20, 21]; our study shows that the gut resistomes of twin pairs also remain similar to each other throughout the first year of life, reinforcing the influence of shared host genetic and environmental variables driving resistome development. Infant gut resistomes were no more similar to those of their mothers than to unrelated infants, indicating that factors other than the maternal gut microbial composition are critical for shaping the infant fecal microbiome. Recent work has shown that microbial communities on environmental surfaces in hospitals and homes significantly affect the composition of host-associated microbial communities for humans in those habitats [30, 31], and soil microbes have also been found to have resistance genes identical to those in human pathogens [32]. Although antibiotic-resistant bacteria have been identified on many food products [33–35], 1–2-month-old infants are unlikely to have substantial solid food exposure, and thus environmental surface exposures are more likely to shape the gut resistome of young infants. The association between resistance genes that are shared within and between families suggests a potential role for surveillance of resistance genes within families as a first indicator of resistance genes that are becoming prevalent in the larger community. Infants tended to have resistance to clinically important broad-spectrum β-lactam antibiotics that their mothers lacked; this, in combination with the dissimilarity between the populations of infant and maternal β-lactamases, suggests that understanding the factors predisposing infant microbiota to harbor these undesirable β-lactamases may lead to better understanding of the determinants of widespread broad-spectrum β-lactamase resistance in human communities. Finally, we describe a developmental progression for a potentially core microbial function of chloramphenicol resistance, which progresses from Enterobacteriaceae multidrug efflux pumps in early infancy to an adult-like population of chloramphenicol acetyltransferases that are often encoded by cryptic microbes, providing new insight into the development of microbiome functions in the early life of the human host.

## Methods

Maternal stools were collected at delivery, and infant fecal samples were collected at 1–2 (baseline), 6–7 (30 days after solid food initiation), and 11 months of age (post-antibiotic exposure). All fecal samples used in this study were collected with informed consent for the St. Louis Neonatal Microbiome Initiative (P.I. Dr. Barbara Warner). This study and the St. Louis Neonatal Microbiome Initiative were approved by Washington University’s Institutional Review Board (IRB# 201205152 and 201105492, respectively). Demographics and clinical metadata were securely stored in a RedCap [36] database. The resistomes encoded by these infant and maternal fecal samples were characterized using functional metagenomic selections [4, 5, 32]. We constructed metagenomic libraries by shotgun cloning one- to five-Kb DNA fragments from the 21 fecal samples (Additional file 1: Table S1) into a pZE21 plasmid vector, and transforming the plasmids into *E. coli* as previously described [5]. These libraries were screened for resistance against 18 antibiotics representing eight drug classes (β-lactams, tetracyclines, aminoglycosides, amphenicols, quinolones, sulfonamides, polymyxins, and cycloserine). Resistance was observed to 16 of the 18 antibiotics tested (Additional file 1: Table S2); resistance-conferring fragments were sequenced, assembled, and annotated using the Parallel Annotation and Reassembly of Functional Metagenomic Selections (PARFuMS) iterative assembly pipeline [32] (see online methods). Predicted proteins were clustered at the 97 % identity level using cd-hit [17, 18]. Resistance proteins and mobilization elements were identified by a previously described keyword string search of predicted proteins annotated by the PARFuMS computational pipeline [5]. Statistical analysis was performed on these 97 % ID clusters using SAS version 9.3. Analyses on resistance protein clusters were performed using log-linear models assuming a negative binomial distribution of protein cluster counts. Tables of pairwise sample similarity between all samples were calculated using the binary Jaccard distance metric from the QIIME package [37].

### Availability of supporting data

The data reported in this paper are tabulated in Additional file 1 and archived in NCBI Genbank (BioProject ID PRJNA287613).

## Additional file

Additional file 1: ** Supplementary materials. Table S1.** Twin demographics. **Table S2.** Antibiotics used for functional selections. **Table S3.** Log-linear model of multidrug efflux pump protein clusters identified in beta-lactam selections following amoxicillin exposure. **Table S4–S5.** Log-linear model of within-family and between-family sharing of resistance-associated protein clusters. **Table S6.** Log-linear model of multidrug efflux pump protein clusters identified in chloramphenicol selections in infants and mothers. **Table S7.** Log-linear model of multidrug efflux pump protein clusters identified in chloramphenicol selections in infants over time. **Table S8–S9.** Log-linear model of chloramphenicol acetyltransferase protein clusters identified in chloramphenicol selections in infants over time. **Table S10.** Log-linear model of within-family sharing, between-family sharing, and appearance of resistance-associated protein clusters at difference points in time. **Figure S1.** Antibiotic resistance phenotypes. **Figure S2.** Beta-lactam resistance genotype and phenotype. **Figure S3.** Twin infant fecal resistomes resemble those of their siblings at each timepoint.

## Abbreviations

Gb: gigabases
kb: kilobases
mgDNA: metagenomic DNA
PARFuMS: parallel annotation and reassembly of functional metagenomic selections
