# Erratum: Gut resistome development in healthy twin pairs in the first year of life

**DOI:** 10.1186/s40168-015-0095-4

**Published:** 2015-07-23

**Authors:** Aimee M. Moore, Sara Ahmadi, Sanket Patel, Molly K. Gibson, Bin Wang, I. Malick Ndao, Elena Deych, William Shannon, Phillip I. Tarr, Barbara B. Warner, Gautam Dantas

**Affiliations:** Department of Pediatrics, Washington University in St Louis School of Medicine, 660 S. Euclid Avenue, St. Louis, MO 63110 USA; Department of Pathology and Immunology, Washington University in St. Louis School of Medicine, 660 S. Euclid Avenue, St. Louis, MO 63110 USA; Center for Genome Sciences and Systems Biology, Washington University in St. Louis School of Medicine, 4444 Forest Park Boulevard, St. Louis, MO 63108 USA; Department of Biostatistics, Washington University in St. Louis School of Medicine, 660 S. Euclid Avenue, St. Louis, MO 63110 USA; Department of Molecular Microbiology, Washington University in St. School of Medicine, 660 S. Euclid Avenue, St. Louis, MO 63110 USA; Department of Biomedical Engineering, Washington University in St. Louis, One Brookings Drive, St. Louis, MO 63130 USA

In our original published manuscript entitled “Gut resistome development in healthy twin pairs in the first year of life” [1], the legends for Figures 4 and 5 were incorrectly transposed.

The correct legend for Figure 4 should read “β-lactam phylogenetic tree, annotated by study subject. Maternal subjects are marked with an “M”. Infant fecal samples are marked with a number; “1” indicating the first (baseline) sample collected at 1–2 months of age, “2” indicating the second sample collected at 6–7 months of age, and “3” indicating the third sample collected at 11 months of age. The antibiotic-naïve control family is colored *green*, the family with infants discordant for amoxicillin exposure at 8 months of age is colored *purple*, and the family with infants concordant for amoxicillin exposure at 8 months of age is colored *brown*. Infant twin A subjects are *shaded darker*; twin B subjects are *shaded lighter*. β-lactamases were commonly present in both members of a twin pair, and frequently persisted at more than one timepoint within a given subject. Many β-lactamases identified in the infant fecal microbiomes were not present in the maternal microbiome.”

The correct legend for Figure 5 should read “Populations of chloramphenicol resistance proteins change over time. Predicted proteins found when fecal metagenomic libraries were screened on chloramphenicol-containing media were collapsed into 97 % ID clusters. *Black boxes* signify genes encoding a resistance protein that were identified in the fecal metagenome of a study subject at a given timepoint, while *white* or *light gray squares* indicate that the protein was not present. Proteins that were co-localized with a mobile genetic element are marked with an *asterisk*. Chloramphenicol acetyltransferases were found in all mothers and in five of the six infants at the final timepoint, but were qualitatively less common in infants at earlier timepoints. By contrast, multidrug efflux pumps were rare in mothers and in 11-month-old infants, but were commonly found in earlier samples”.

The name of the co-author “I. Malick Ndao” was also incorrectly transposed as “Malick I. Ndao”.

We regret these errors, which have been corrected.
