# Supplementary material for: Gut resistome development in healthy twin pairs in the first year of life
Source: Microbiome. 2015 Jun 25;3:27. doi: 10.1186/s40168-015-0090-9 (PMC4480905; doi:10.1186/s40168-015-0090-9)
Supplement: Supplementary file 1 — Supplementary materials. Table S1. Twin demographics. Table S2. Antibiotics used for functional selections. Table S3. Log-linear model of multidrug efflux pump protein clusters identified in beta-lactam selections following amoxicillin exposure. Table S4–S5. Log-linear model of within-family and between-family sharing of resistance-associated protein clusters. Table S6. Log-linear model of multidrug efflux pump protein clusters identified in chloramphenicol selections in infants and mothers. Table S7. Log-linear model of multidrug efflux pump protein clusters identified in chloramphenicol selections in infants over time. Table S8–S9. Log-linear model of chloramphenicol acetyltransferase protein clusters identified in chloramphenicol selections in infants over time. Table S10. Log-linear model of within-family sharing, between-family sharing, and appearance of resistance-associated protein clusters at difference points in time. Figure S1. Antibiotic resistance phenotypes. Figure S2. Beta-lactam resistance genotype and phenotype. Figure S3. Twin infant fecal resistomes resemble those of their siblings at each timepoint. [file 40168_2015_90_MOESM1_ESM.pdf]

Supplementary Materials.

- I. **Materials and Methods**
  - a. **Fecal Metagenomic Library Construction and Antibiotic Screening**
  - b. **Illumina Library Preparation**
  - c. **PARFuMS Assembly and Annotation**
  - d. **Assignment of Predicted Protein Ontology and Clustering**
  - e. **Collection of Twin Demographic Information**
- II. **Supplementary Results**
  - a. **Metagenomic Library Phenotypes**
  - b. **Beta-Lactam Resistance Genes**
- III. **Tables S1-S9**
- IV. **Figures S1-S2**

I. Materials and Methods

a. Fecal Metagenomic Library Construction and Antibiotic Screening

All fecal samples used in this study were collected with informed consent for the St. Louis Neonatal Microbiome Initiative (P.I. Dr. Barbara Warner). This study and the St. Louis Neonatal Microbiome Initiative were approved by Washington University's Institutional Review Board (IRB# 201205152 and 201105492, respectively). Demographics and clinical metadata were securely stored in a RedCap(*1*) database. Metagenomic DNA (mgDNA) was extracted from frozen fecal samples as previously described(*2*). MgDNA was sheared using the Covaris M220 (Shearing conditions as follows: 2-20ug metagenomic DNA were diluted to a volume of 200uL in Buffer EB. Settings were: duty cycle 20%, intensity 0.1, cycles per burst 1,000, and total treatment time 600s). Metagenomic libraries were prepared as previously described(*3, 4*): sheared DNA fragments 2-5kb in size were selected by gel electrophoresis in a 1% low-melting point agarose gel in 0.5X Tris-Borate-EDTA buffer stained with GelGreen dye (Biotium). The fragments were purified using a QIAquick Gel Extraction kit (Qiagen), and end-repaired with the END-It DNA End Repair Kit (Epicentre). End-repaired fragments were purified with the QIAquick PCR purification kit (Qiagen), quantitated with the Qbit HS fluorometer (Invitrogen), and ligated with the Fast Link Ligation kit (Epicentre) at the HincII site to a pZE21 plasmid vector that was linearized by PCR amplification as previously described(*4*). Ligation reactions were dialyzed with a cellulose membrane (Millipore VSWP09025) and electroporated into *E. coli* MegaX DH10B T1R (Invitrogen) to create metagenomic libraries. These metagenomic libraries were amplified in Luria-Bertani broth and library titers determined by serial dilution as previously described(*3*). Amplified libraries were screened on Mueller-Hinton agar plates with kanamycin (50ug/mL) and one of 18 antibiotics representing 8 drug classes (Table S2). For each screening plate, the concentration of the library was adjusted such that the number of clones

plated was equal to 10x the number of unique clones in the library. For each selection condition, a control organism (MegaX DH10B T1R transformed with unmodified pZE21) was also screened to confirm susceptibility of the unmodified host organism.

b. Illumina library preparation

Metagenomic inserts from antibiotic-resistant clones were prepared for Illumina sequencing exactly as previously described(*4*). Colonies that grew on antibiotic screening plates were scraped into a slurry with a solution of 15% glycerol in Luria-Bertani broth and frozen at -80C prior to further processing. Aliquots of plate-scrape slurries underwent cell lysis via centrifugation and freeze-thaw, insoluble solids were pelleted via centrifugation, and the supernatant was used as a template for PCR amplification of functionally-selected resistance-conferring DNA fragments(*4*) using Taq polymerase (New England Biolabs) and a custom mix of primers designed to complement DNA flanking the HincII insertion site (primer F1, 5′-CCGAATTCATTAAAGAGGAGAAAG; primer F2, 5′-CGAATTCATTAAAGAGGAGAAAGG; primer F3, 5′-GAATTCATTAAAGAGGAGAAAGGTAC; primer R1, 5′-GATATCAAGCTTATCGATACCGTC; primer R2, 5′-CGATATCAAGCTTATCGATACCG; primer R3, 5′-TCGATATCAAGCTTATCGATACC) . The amplified resistance-conferring fragments were purified using the QIAquick PCR purification kit, quantitated using the Qubit fluorometer, and sheared using the BioRuptor XL as previously described(*4*). Sheared DNA was purified with the QIAGEN MinElute PCR Purification kit, then end-repaired with Taq polymerase, T4 polymerase, T4 PNK, and dNTPs as previously described.(*4*) Pre-annealed barcoded sequencing adapters were added to each end-repaired sample with a unique 7bp oligonucleotide sequence assigned to each library and antibiotic selection combination as previously described.(*4*) Barcoded samples were purified using the Qiagen PCR Purification kit and size-selected for 300-400bp fragments on a 2% agarose gel in 0.5X TBE, then purified using a QIAGEN MinElute Gel Extraction kit. The purified size-selected barcoded DNA was PCR-enriched using Illumina PCR Primer Mix and Phusion HF MasterMix as previously described.(*4*) PCR-enriched samples were pooled for sequencing (10nM/sample) and submitted for Illumina Hi-Seq paired-end 101-bp sequencing using the HiSeq 2000 platform at Washington University's GTAC (Genome Technology Access Center, St Louis, Missouri, USA).

c. PARFuMS Assembly and Annotation

Functionally-selected metagenomic fragments submitted for Illumina platform sequencing were assembled and annotated as previously described.(*4*) Paired-end sequence reads were binned by exact-match barcodes, and fragments corresponding to each selection condition were assembled and annotated using the PARFuMS (Parallel Annotation and Re-assembly of Functional Metagenomic Selections) tool(*4, 5*). Fragments were assembled in an iterative process using Velvet(*6*), Phrap(*7*), and custom scripts as previously described.(*4*) Open reading frames (ORFs)

were identified using MetaGeneMark(*8*), and annotated by using HMMER3(*9*) to search against the TIGRFAMS(*10*) and PFam(*11*) databases.

d. Assignment of Predicted Protein Ontology and Clustering

Predicted protein ontologies were assigned to four categories "resistance gene", "mobilization element", "transporter/efflux pump", and "other" according to a keyword string search of the annotations as previously described (*3*). Predicted proteins were clustered at the 97% identity level using Cd-hit(*12*) and contigs <500bp in size were filtered out. Statistical analysis was performed on these 97% ID clusters using SAS version 9.3. Analyses were performed using log-linear models assuming a negative binomial distribution of protein cluster counts. For analyses of repeated measurements (months 6 and 11) the models were adjusted for within-subject and within-family correlation. Tables of Pairwise sample similarity (1-dissimilarity) between all samples were calculated using the binary Jaccard distance metric using the *beta_diversity.py* script from the QIIME package(*13*). Significance of the groups in Figure 1 was calculated using the *all_pairs_t_test* function from the QIIME(*13*) stats module.

e. Collection of Twin Demographic Information

Infant diets were ascertained via parental questionnaire; antibiotic exposures were also reported via parental questionnaire and verified in the infants' medical records from their primary care pediatricians.

II. Supplementary Results

a. Metagenomic Library Phenotypes

The gut microbial communities of all subjects at all time points harbored genes conferring resistance to a broad array of antibiotics (Fig. S1). As in our previous study of the pediatric fecal resistome, resistance to chloramphenicol, tetracycline, cycloserine, and sulfonamides (trimethoprim alone and in combination with sulfamethoxazole) was universal, suggesting that these are key components of human-associated fecal resistomes. Resistance to colistin, tigecycline, and gentamicin were less frequently observed. There were no apparent time-dependent trends observed in infant resistome phenotypes during the first year of life: all infant gut metagenomes demonstrated resistance to multiple antibiotics at the initial time point (1-2 months of age), and this resistance pattern tended to persist throughout the first year. There were no apparent effects of amoxicillin exposure on the resistance phenotype, again suggesting that the fecal resistome is established early, and is robust in the face of antibiotic perturbation at 8 months of age.

b. Beta-Lactam Resistance Genes

In the study cohort, all classes of beta-lactamases were represented. There were two major groups of Class A beta-lactamases, including one group with high identity to TEM beta-

lactamases that was associated with extended-spectrum penicillin and beta-lactamase inhibitor resistance, and another group of class A beta-lactamases that were more closely associated with later-generation cephalosporin resistance (Figs. 3, S2). All fourth-generation cephalosporin resistance was associated with either Class A or Class D beta-lactamases. The cephalosporin-resistant group included a novel beta-lactamase (CL1135), which had only 67% protein identity to a beta-lactamase from Akkermansia CAG:344 (WP_022395938); the source contig was dissimilar to any known sequence, with only 76% identity to Akkermansia muciniphilia (CP001071). This beta-lactamase was found in third- and fourth-generation cephalosporin selections, emphasizing the potential for poorly-understood gut microbes to be a source of resistance to clinically important antibiotics, even ones such as ceftazidime and cefepime, that are not used in the outpatient setting. The TEM-like group of Class A beta-lactamases included three proteins syntenic with mobilization elements, all of which had identical protein sequences to known beta-lactamases from a variety of gut microbes: CL1167 to Bacillus subtilis beta lactamase YP_004205979, CL1069 to Clostridium Boltae beta-lactamase WP_002567442, also found in our survey of the resistomes of healthy children, and CL1191 to Selenomonadales beta lactamase WP_006555379, which was found only in infants and was associated with third- and fourth-generation cephalosporin and monobactam resistance. The TEM-like group also included beta-lactamases identical to proteins identified in human pathogens: CL1172 to klebsiella pneumoniae TEM beta-lactamase WP_004151611 and CL1114 to Klebsiella oxytoca WP_004137840, both of which were found only in infants and were found in extended-spectrum penicillin selections, both with and without beta-lactamase inhibitors. This data is in agreement with our previous finding that highly undesirable extended-spectrum beta-lactamases are often found in healthy infants

Class C beta-lactamases were found in all subjects, and primarily had high identity to Escherichia coli or Enterobacter aerogenes ampC beta-lactamases. Classes B and D were much less common, with only two Class B beta-lactamases found, both in mothers. Class D beta-lactamases included two novel beta-lactamases, one (CL1013) that had only 56% protein identity to a clostridial beta-lactamase (WP_005837179) and a second (CL1169) that was syntenic with a mobile element and had only 70% protein identity to a class D beta-lactamase from Firmicutes CAG:114 (WP_021920041). In both cases, the source contigs had only short-segment identity to any known sequences (CL1013 with 69% identity and 11% coverage to clostridium saccharolyticum (FP929037) and CL1169 with 84% identity over only 9% coverage to Eubacterium rectale (CP001107)). This underscores the potential importance of cryptic gut microbes as a source of novel, mobilizable resistance genes.

Family ID	Twin ID	Gender	Placentation	Zygosity	Birth weight (g)	Gestational Age (weeks)	Route of delivery	Maternal Intrapartum Antibiotics
Amoxicillin Discordant	A	Female	Dichorionic	Unknown	3013	38	Vaginal	None
	B	Female	Dichorionic	Unknown	3008	38	Vaginal	None
Antibiotic Naïve	A	Male	Dichorionic	Dizygotic	2768	37	Vaginal	ampicillin-sulbactam
	B	Male	Dichorionic	Dizygotic	3041	37	Vaginal	ampicillin-sulbactam
Amoxicillin Concordant	A	Male	Dichorionic	Monozygotic	2450	36	Vaginal	ampicillin
	B	Male	Dichorionic	Monozygotic	2445	36	Vaginal	ampicillin

Table S1. Twin Demographics

Antibiotic Class		Antibiotic	Concentration
Beta Lactam	Penicillin	Penicillin	128 ug/mL
	Extended-spectrum	Piperacillin	16 ug/mL
	+ beta-lactamase inhibitor	Piperacillin-Tazobactam	16/4 ug/mL
	2nd generation cephalosporin	cefoxitin	32 ug/mL
	3rd generation cephalosporin	cefotaxime	8 ug/mL
		ceftazidime	16 ug/mL
	4th generation cephalosporin	cefepime	8 ug/mL
	carbapenem	meropenem	16 ug/mL
	monobactam	aztreonam	8 ug/mL
Aminoglycoside		gentamicin	16 ug/mL
Tetracyclines		tetracycline	8 ug/mL
		Tigecycline	2 ug/mL
Polymyxin		colistin	8 ug/mL
Quinolone		ciprofloxacin	0.5 ug/mL
Folate synthesis inhibitors		trimethoprim	8 ug/mL
		Trimethoprim-sulfamethoxazole	2/38 ug/mL
cycloserine		D-cycloserine	32 ug/mL
amphenicol		chloramphenicol	8 ug/mL

Table S2. Antibiotics used for functional selections. All antibiotic selections were performed in Mueller-Hinton agar with 50ug/mL kanamycin.

II. MDR_in_Bla_Sel=1							
**Empirical Standard Error Estimates**							
**Parameter**		**Estimate**	**Standard Error**	**95% Confidence Limits**		**Z**	**Pr > |Z|**
**Intercept**		-0.5469	0.6788	-1.8774	0.7836	-0.81	0.4205
**time**	11	0.8196	0.4895	-0.1399	1.7790	1.67	0.0941
**time**	6	0.0000	0.0000	0.0000	0.0000	.	.
**AbxExp**	0	2.6053	0.5800	1.4686	3.7420	4.49	<.0001
**AbxExp**	1	0.0000	0.0000	0.0000	0.0000	.	.
**mobl**		-4.6687	0.9088	-6.4500	-2.8874	-5.14	<.0001

Table S3. Log-linear model of multidrug efflux pump protein clusters identified in beta-lactam selections following amoxicillin exposure. The count matrix in the model includes family ID (Control = antibiotic-naïve control, Abx Disc = twins discordant for antibiotic exposure, Abx Conc = twins concordant for antibiotic exposure), twin ID (1 = twin A, 2 = twin B), timepoint at which the source fecal sample was collected (6 or 7 months, and 11 months), exposure to antibiotics prior to collection of the source fecal sample (AbxExp 0 = no antibiotic exposure; AbxExp 1 = exposure to amoxicillin), co-localization with a mobilization element (mobl 0 = no co-localization, mobl 1 = co-localization), and the number of multidrug efflux pumps identified under the conditions stipulated. The output table describes the contributions of three variables (time, antibiotic exposure, and co-localization with a mobilization element) to the model. Multidrug efflux proteins were significantly more likely to be found in beta-lactam selections following antibiotic exposure. They were significantly less likely to be co-localized with a mobilization element than not.

Analysis Of Maximum Likelihood Parameter Estimates							
Parameter	DF	Estimate	Standard Error	Wald 95% Confidence Limits		Wald Chi-Square	Pr > ChiSq
Intercept	1	11.9656	0.8974	10.2068	13.7244	177.80	<.0001
FamShare	1	-3.3731	0.4639	-4.2822	-2.4639	52.87	<.0001
MaxShare	1	-3.2655	0.4561	-4.1594	-2.3716	51.27	<.0001
FamShare*MaxShare	1	1.1797	0.2281	0.7326	1.6268	26.74	<.0001
Dispersion	1	0.1029	0.0768	0.0238	0.4440		

Table S4. Log-linear model of within-family and between-family sharing of resistance-associated protein clusters. The count matrix in the model includes the number of families a protein cluster was found in (FamShare: 1, 2, or 3 families), the number of members of a given family a protein cluster was found in (MaxShare: 1, 2, or 3 members),and the number of resistance protein clusters identified under the conditions stipulated. The output table describes the contributions of three variables (FamShare, MaxShare, and an interaction term FamShare*MaxShare) to the model. Resistance proteins were significantly less likely to be found in multiple members of the same family or in multiple families, but there was a significant positive interaction between within-family and between-family sharing (proteins found in more than one member were more likely to also be found in more than one family.

Analysis Of Maximum Likelihood Parameter Estimates							
**Parameter**	**DF**	**Estimate**	**Standard**	**Wald 95% Confidence**		**Wald Chi-Square**	**Pr > ChiSq**
**Intercept**	1	8.1273	0.6879	6.7790	9.4756	139.58	<.0001
**FamShare**	1	-1.1041	0.3124	-1.7164	-0.4918	12.49	0.0004
**Mobile**	1	-3.7332	1.0438	-5.7791	-1.6873	12.79	0.0003
**FamShare*Mobile**	1	0.5502	0.4941	-0.4183	1.5186	1.24	0.2655
**MaxShare**	1	-1.2461	0.3389	-1.9102	-0.5819	13.52	0.0002
**Mobile*MaxShare**	1	0.6047	0.4943	-0.3642	1.5735	1.50	0.2212
**Dispersion**	1	0.1029	0.0768	0.0238	0.4440		

Table S5. Log-linear model of within-family and between-family sharing of resistance-associated protein clusters. The count matrix in the model includes the number of families a protein cluster was found in (FamShare: 1, 2, or 3 families), the number of members of a given family a protein cluster was found in (MaxShare: 1, 2, or 3 members), co-localization with a mobilization element (Mobile = 1: co-localization with a mobile element; Mobile = 0: no co-localization), and the number of resistance protein clusters identified under the stipulated conditions. The output table describes the contributions of five variables (FamShare, MaxShare, Mobile and the interaction terms FamShare*Mobile and MaxShare*Mobile) to the model. There was no significant positive association between co-localization with a mobilization element and sharing within (MaxShare) or between (FamShare) familes.

Analysis Of GEE Parameter Estimates							
Empirical Standard Error Estimates							
**Parameter**		**Estimate**	**Standard Error**	**95% Confidence Limits**		**Z**	**Pr > |Z|**
**Intercept**		1.7346	0.2553	1.2343	2.2349	6.79	<.0001
**Mom**		-2.8332	0.8555	-4.5099	-1.1565	-3.31	0.0009

Table S6. Log-linear model of multidrug efflux pump protein clusters identified in chloramphenicol selections in infants and mothers. The model includes columns for family ID (Control = antibiotic-naïve control, Abx Disc = twins discordant for antibiotic exposure, Abx Conc = twins concordant for antibiotic exposure), member ID (0= mother, 1 = twin A, 2 = twin B), and the number of multidrug efflux pumps identified under the conditions stipulated. The output table describes the contributions of the variable "mom" (member ID = 0) to the model. Multidrug efflux proteins were significantly more likely to be found in infants than in mothers.

Empirical Standard Error Estimates							
Parameter		Estimate	Standard Error	95% Confidence Limits		Z	Pr > |Z|
Intercept		1.5044	0.2755	0.9643	2.0445	5.46	<.0001
Month		-0.1880	0.0294	-0.2456	-0.1304	-6.40	<.0001

Table S7. Log-linear model of multidrug efflux pump protein clusters identified in chloramphenicol selections in infants over time. The count matrix in the model includes for family ID (Control = antibiotic-naïve control, Abx Disc = twins discordant for antibiotic exposure, Abx Conc = twins concordant for antibiotic exposure), member ID (1 = twin A, 2 = twin B), timepoint (1, 6-7, and 11 months), and the number of multidrug efflux pumps identified under each condition. The output table describes the contributions of the variable "month" to the model. Multidrug efflux proteins were significantly less likely to be found at later timepoints.

Analysis Of GEE Parameter Estimates							
Empirical Standard Error Estimates							
**Parameter**		**Estimate**	**Standard Error**	**95% Confidence Limits**		**Z**	**Pr > |Z|**
**Intercept**		0.5108	0.1826	0.1530	0.8687	2.80	0.0051
**Mom**		0.8755	0.1826	0.5176	1.2333	4.80	<.0001

Table S8. Log-linear model of chloramphenicol acetyltransferase protein clusters identified in chloramphenicol selections in infants over time. The count matrix in the model includes family ID (Control = antibiotic-naïve control, Abx Disc = twins discordant for antibiotic exposure, Abx Conc = twins concordant for antibiotic exposure), member ID (0 = mom, 1 = twin A, 2 = twin B), and the number of chloramphenicol acetyltransferases identified under each condition. The output table describes the contributions of the variable "mom" (member ID = 0) to the model. Chloramphenicol acetyltransferases were significantly more likely to be found in mothers than in infants.

Empirical Standard Error Estimates							
Parameter		Estimate	Standard Error	95% Confidence Limits		Z	Pr > |Z|
Intercept		-1.1785	0.7206	-2.5907	0.2338	-1.64	0.1019
Month		0.0866	0.0824	-0.0748	0.2481	1.05	0.2930

Table S9. Log-linear model of chloramphenicol acetyltransferase protein clusters identified in chloramphenicol selections in infants over time. The count matrix in the model includes family ID (Control = antibiotic-naïve control, Abx Disc = twins discordant for antibiotic exposure, Abx Conc = twins concordant for antibiotic exposure), member ID (1 = twin A, 2 = twin B), and timepoint (1, 6-7, and 11 months), and number of chloramphenicol acetyltransferases identified under each condition. The output table describes the contributions of the variable "month" to the model. Chloramphenicol acetyltransferases were not significantly more likely to be found at later timepoints.

Analysis Of Maximum Likelihood Parameter Estimates							
Parameter	DF	Estimate	Standard Error	Wald 95% Confidence Limits		Wald Chi-Square	Pr > ChiSq
Intercept	1	11.6053	0.6307	10.3691	12.8414	338.57	<.0001
FamShare	1	-3.2214	0.3376	-3.8832	-2.5596	91.03	<.0001
MaxShare	1	-3.1086	0.3294	-3.7543	-2.4629	89.04	<.0001
FamShare*MaxShare	1	1.0810	0.1672	0.7533	1.4087	41.80	<.0001
SelfDiffTime	1	-2.8207	0.5071	-3.8146	-1.8268	30.94	<.0001
MaxShare*SelfDiffTime	1	0.5487	0.2736	0.0124	1.0849	4.02	0.0449
Dispersion	1	0.0541	0.0489	0.0092	0.3183		

Table S10. Log-linear model of within-family sharing, between-family sharing, and appeareace of resistance-associated protein clusters at different points in time. The count matrix in the model includes the number of families a protein cluster was found in (FamShare: 1, 2, or 3 families), the number of members of a given family a protein cluster was found in (MaxShare: 1, 2, or 3 members), whether or not a resistance protein cluster was found in an individual at multiple timepoints (SelfDiffTime; 1= present at multiple timepoints; 0 = present at a single timepoint), and the number of resistance protein clusters identified under the stipulated conditions. The output table describes the contributions of five variables (FamShare, MaxShare, SelfDiffTime and the interaction terms FamShare*MaxShare and MaxShare*SelfDiffTime) to the model. There were significant positive associations between sharing within (MaxShare) or between (FamShare) familes and between sharing within families (MaxShare) and persistence within an individual at different timepoints (SelfDiffTime). Models including interaction terms between persistence at different timepoints and sharing between families (SelfDiffTIme*FamShare) or sharing within and between families (SelfDiffTime*FamShare*MaxShare) did not show any significant contributions from those interaction terms.

	Antibiotic-Naïve Control							Amoxicillin Discordant							Amoxicillin Concordant						
		**Twin A**			**Twin B**				**Twin A**			**Twin B**				**Twin A**			**Twin B**		
	**M**	**1**	**2**	**3**	**1**	**2**	**3**	**M**	**1**	**2**	**3**	**1**	**2**	**3**	**M**	**1**	**2**	**3**	**1**	**2**	**3**
**Penicillin**	■	■	■	■	■	■	■	■	■	■	■	■	■	■	■	■	■	■	■	■	■
**Piperacillin**		■	■	■	■	■	■	■	■		■	■	■	■		■	■	■	■	■	■
**Piperacillin-Tazobactam**		■	■	■		■		■	■		■	■		■		■	■	■	■	■	■
**Cefoxitin**	■	■	■	■	■	■	■	■	■		■		■		■	■			■		
**Cefotaxime**	■	■	■	■	■	■	■		■		■	■	■		■	■					
**Ceftazidime**	■	■	■	■	■	■	■	■	■	■	■	■	■	■	■				■	■	■
**Cefepime**			■	■	■	■	■														
**Aztreonam**	■	■	■	■	■	■	■	■		■						■			■		
**Meropenem**																					
**Tetracycline**	■	■	■	■	■	■	■	■	■	■	■	■	■	■	■	■	■	■	■	■	■
**Tigecycline**													■		■						
**Trimethoprim**	■	■	■	■	■	■	■	■	■	■	■	■	■	■	■	■	■	■	■	■	■
**Trimethoprim-Sulfamethoxazole**	■	■	■	■	■	■	■	■	■	■	■	■	■	■	■	■					
**Gentamicin**	■							■			■	■		■	■						■
**Chloramphenicol**	■	■	■	■	■	■	■	■	■	■	■	■	■	■	■	■	■	■	■	■	■
**Colistin**	■		■	■		■	■								■						
**Cycloserine**	■	■	■	■	■	■	■	■	■	■	■	■	■	■	■	■	■	■	■	■	■
**Ciprofloxacin**																					
**Library Size**	2.1	20	14.5	24.3	24.1	24.1	6.5	16.6	6.1	4.3	2.7	10.3	10	6.3	3.13	1.8	4.6	3.3	2.5	6.3	2.4

Fig. S1. Antibiotic Resistance Phenotypes. Metagenomic libraries were constructed from fecal samples collected from three mother-twin triads. One twin pair was antibiotic-naïve for the 11-month study period, and one pair was concordant and the other discordant for a 10-day course of oral amoxicillin administered at 8 months of age. Maternal libraries were constructed using fecal samples collected at the time of delivery. Infant libraries were constructed from fecal samples collected at three timepoints: 1-2 months of age (baseline), 6-7 months of age (30 days after solid food initiation), and 11 months of age (30 days following antibiotic exposure). The metagenomic libraries were screened against an array of antibiotics that were inhibitory to the control organism. Libraries that demonstrated resistance to a given antibiotic are represented by dark squares; if the antibiotic screening plates had no growth, then there is a corresponding white square. There was no resistance to meropenem or ciprofloxacin in any of the subjects. All subjects at all time points had resistance to multiple antibotics.

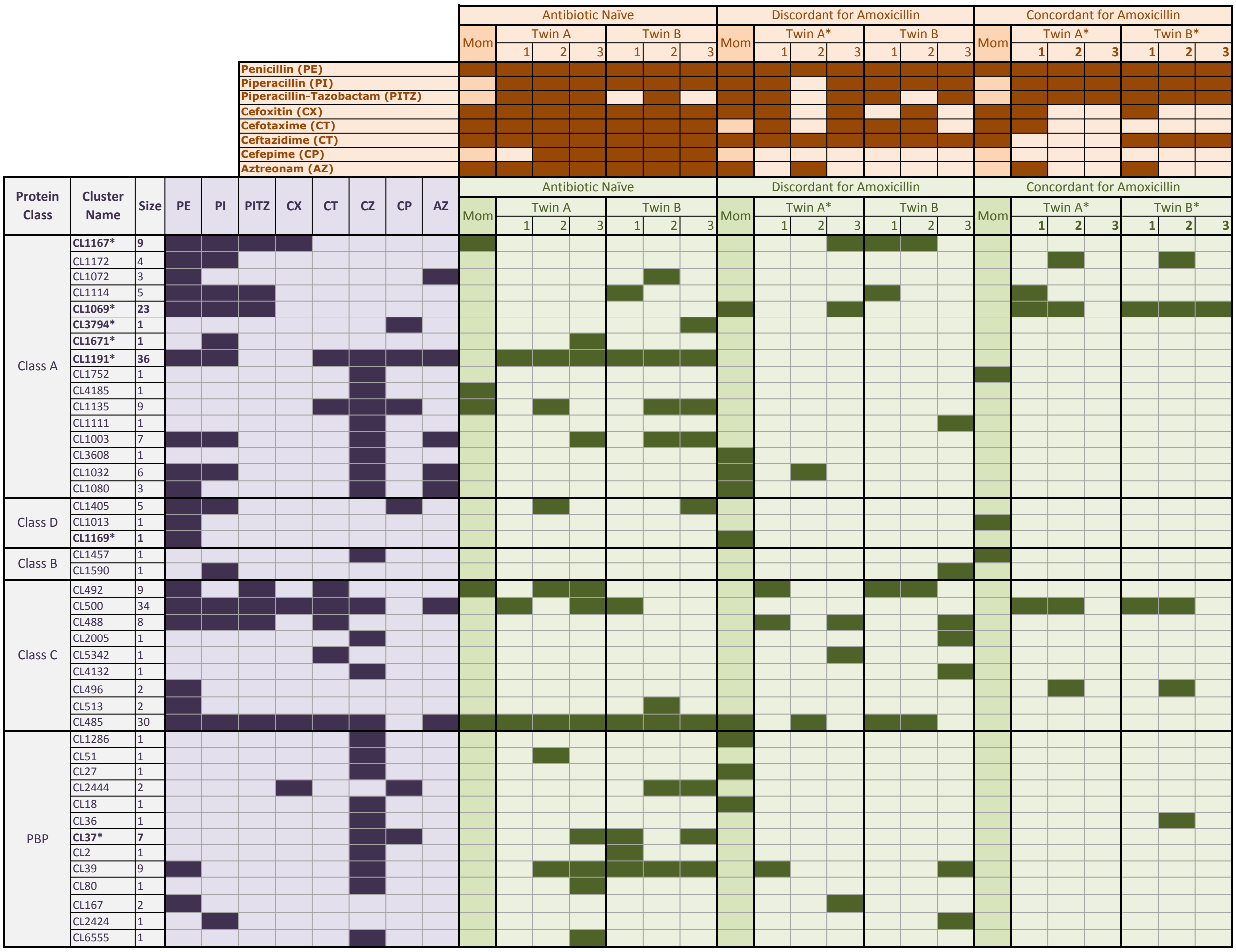

Fig. S2. Beta-Lactam Resistance Genotype and Phenotype. Predicted beta-lactam resistance proteins, collapsed into 97% identity clusters, are sorted by class and ordered according to position on a phylogenetic tree (Fig 5). Antibiotic selection conditions are shaded purple, with dark purple indicating that a resistance protein was identified in that antibiotic selection condition, and a light purple square indicating that the resistance protein was not identified in that condition. Class A beta-lactamases fall into two distinct groups; one with resistance to extended-spectrum penicillins and beta-lactamase inhibitors, and one with resistance to cephalosporins. Class C beta-lactamases highlighted cephalosporin resistance. All Cefepime resistance was found in class A or D beta-lactamases or penicillin binding proteins. Study subjects are shaded green; dark green indicates that a given resistance protein was identified in a given subject at a given timepoint. Many beta-lactam resistance proteins persist over time (are present within a given individual at multiple timepoints) or are present in both infants in a sibling pair. Mothers and infants tend to have different sets of beta-lactam resistance proteins. The resistance phenotypes associated with the fecal metagenome of each study subject is highlighted orange, with dark orange indicating that resistance to that antibiotic was observed at that timepoint.

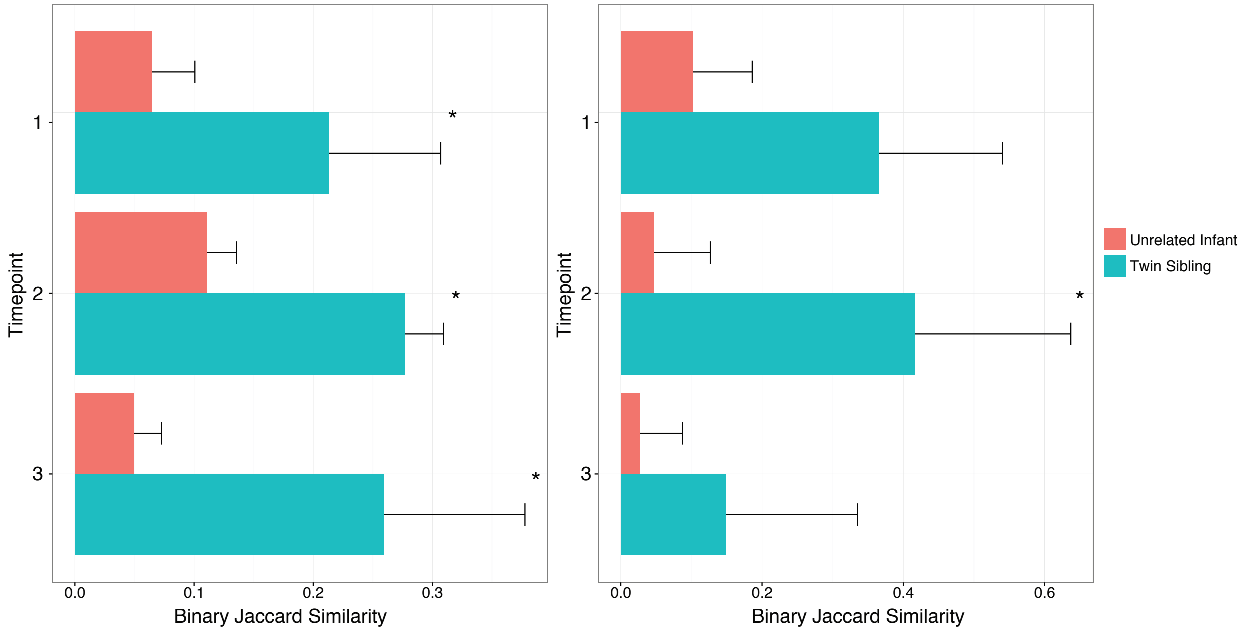

Fig. S3. Twin Infant Fecal Resistomes Resemble Those of their Siblings At Each Timepoint. Predicted resistance proteins were collapsed into 97% identity clusters. Binary Jaccard resistance protein cluster composition similarity was determined at each of the three timepoints for (1) infants and their twin sibling and (2) infants and an unrelated infant sampled at the same timepoint. The panel at left shows all resistance proteins; the panel at right shows the subset of β-lactamases and penicillin binding proteins. Significance was calculated using the Student's t-test with 1,000 Monte Carlo simulations (* $P<0.005$). Infant resistomes overall (panel A) were significantly more similar to a twin sibling or at each timepoint than to those of unrelated infants. With the subset of β-lactamases and penicillin binding proteins, the difference in sharing between twins and their sibling was only significant at the second timepoint, likely due to the smaller sample size

1. P. A. Harris *et al.*, Research electronic data capture (REDCap)--a metadata-driven methodology and workflow process for providing translational research informatics support. *J Biomed Inform* **42**, 377-381 (2009).
2. A. L. Goodman *et al.*, Extensive personal human gut microbiota culture collections characterized and manipulated in gnotobiotic mice. *Proc Natl Acad Sci U S A* **108**, 6252-6257 (2011).
3. A. M. Moore *et al.*, Pediatric Fecal Microbiota Harbor Diverse and Novel Antibiotic Resistance Genes. *PLOS One* **8**, e78822 (2013).
4. K. J. Forsberg *et al.*, Bacterial phylogeny structures soil resistomes across habitats. *Nature* **509**, 612-616 (2014).
5. K. J. Forsberg *et al.*, The shared antibiotic resistome of soil bacteria and human pathogens. *Science* **337**, 1107-1111 (2012).
6. D. R. Zerbino, E. Birney, Velvet: algorithms for de novo short read assembly using de Bruijn graphs. *Genome Res* **18**, 821-829 (2008).
7. M. de la Bastide, W. R. McCombie, Assembling genomic DNA sequences with PHRAP. *Curr Protoc Bioinformatics* **Chapter 11**, Unit11.14 (2007).
8. W. Zhu, A. Lomsadze, M. Borodovsky, Ab initio gene identification in metagenomic sequences. *Nucleic Acids Res* **38**, e132 (2010).
9. R. D. Finn, J. Clements, S. R. Eddy, HMMER web server: interactive sequence similarity searching. *Nucleic Acids Res* **39**, W29-37 (2011).
10. D. H. Haft *et al.*, TIGRFAMs: a protein family resource for the functional identification of proteins. *Nucleic Acids Res* **29**, 41-43 (2001).
11. A. Bateman *et al.*, The Pfam protein families database. *Nucleic Acids Res* **28**, 263-266 (2000).
12. W. Li, A. Godzik, Cd-hit: a fast program for clustering and comparing large sets of protein or nucleotide sequences. *Bioinformatics* **22**, 1658-1659 (2006).
13. J. G. Caporaso *et al.*, QIIME allows analysis of high-throughput community sequencing data. *Nat Methods* **7**, 335-336 (2010).
